# Stress-Induced *In Vivo* Recruitment of Human Cytotoxic Natural Killer Cells Favors Subsets with Distinct Receptor Profiles and Associates with Increased Epinephrine Levels

**DOI:** 10.1371/journal.pone.0145635

**Published:** 2015-12-23

**Authors:** Marc B. Bigler, Simon B. Egli, Cédric M. Hysek, Gideon Hoenger, Laurent Schmied, Fabian S. Baldin, Florian A. Marquardsen, Mike Recher, Matthias E. Liechti, Christoph Hess, Christoph T. Berger

**Affiliations:** 1 Translational Immunology, Dep. of Biomedicine, University Hospital Basel, Basel, Switzerland; 2 Clinical Pharmacology, Dep. of Internal Medicine, University Hospital Basel, Basel, Switzerland; 3 Immunobiology Lab, Dep. of Biomedicine, University Hospital Basel, Basel, Switzerland; 4 Immunotherapy Laboratory, Department of Biomedicine, University Hospital Basel, Basel, Switzerland; 5 Immunodeficiency Lab, Dep. of Biomedicine, University Hospital Basel, Basel, Switzerland; 6 Medical Outpatient Clinic, Dep. of Internal Medicine, University Hospital Basel, Basel, Switzerland; INSERM- CNRS- Univ. Méditerranée, FRANCE

## Abstract

**Background:**

Acute stress drives a ‘high-alert’ response in the immune system. Psychoactive drugs induce distinct stress hormone profiles, offering a sought-after opportunity to dissect the *in vivo* immunological effects of acute stress in humans.

**Methods:**

3,4-methylenedioxymethamphetamine (MDMA), methylphenidate (MPH), or both, were administered to healthy volunteers in a randomized, double-blind, placebo-controlled crossover-study. Lymphocyte subset frequencies, natural killer (NK) cell immune-phenotypes, and changes in effector function were assessed, and linked to stress hormone levels and expression of CD62L, CX3CR1, CD18, and stress hormone receptors on NK cells.

**Results:**

MDMA/MPH > MDMA > MPH robustly induced an epinephrine-dominant stress response. Immunologically, rapid redistribution of peripheral blood lymphocyte-subsets towards phenotypically mature NK cells occurred. NK cytotoxicity was unaltered, but they expressed slightly reduced levels of the activating receptor NKG2D. Preferential circulation of mature NK cells was associated with high epinephrine receptor expression among this subset, as well as expression of integrin ligands previously linked to epinephrine-induced endothelial detachment.

**Conclusion:**

The acute epinephrine-induced stress response was characterized by rapid accumulation of mature and functional NK cells in the peripheral circulation. This is in line with studies using other acute stressors and supports the role of the acute stress response in rapidly mobilizing the innate immune system to counteract incoming threats.

## Introduction

The response of the body to stress consists in the complex integration of endocrine, neuro-cognitive and immunologic adaptations [1]. Chronic persistent stress has been linked to suppressed immune function and increased susceptibility to infections and cancer [1–4], while acute stress induces a fight-or-flight reaction, tuned to rapidly respond to injury and subsequent entry of infectious agents into the organism. Acute stress-induced changes in the immune system thus plausibly aim at accelerating wound repair, help prevent infections, and should occur very rapidly [1].

In contrast to adaptive immunity, the innate immune response is optimized to immediately respond to pathogens without need for prior cognate exposure. Natural Killer (NK) cells represent 5–15% of all lymphocytes in the peripheral blood and are important in the defense against viruses and tumors. Based on their CD56 surface expression-density they can by divided into cytotoxic CD56^dim^ NK cells (roughly 90%), and the immunomodulatory CD56^bright^ NK cells (approximately 10% of NK in the peripheral blood). Circulating cytotoxic CD56^dim^ NK cells detect virally infected or malignantly transformed cells. Their activation results in target cell lysis and the secretion of cytokines [5]. Previous studies found a strong increase of circulating NK cells upon stress, suggesting a potential role for NK cells in this context [6].

Stress hormones, including glucocorticoids and catecholamines, are key modulators of stress-induced immune-dysregulation [7, 8]. However, inter-individual differences in the endocrine responses often hinder subtle stress-associated immunologic alterations to become apparent. Thus, a well-controlled experimental model triggering a homogeneous endocrine stress response should allow to better study how stress impacts the immune system.

3,4-Methylenedioxymethamphetamine (MDMA, “ecstasy”) and methylphenidate (MPH, “Ritalin^®^”) are widely used psychoactive substances that induce an endocrine and cardiovascular stress response that has been extensively studied [9–12]. Previous murine and human studies suggested that MDMA impacts both the innate and the adaptive immune responses [13, 14]. Data regarding the effect of MPH on the immune system are sparse. Both drugs result in an increase in norepinephrine levels, i.e. have sympathomimetic effects. While MDMA enhances serotonergic neurotransmission [15] and increases cortisol levels [11], MPH elevates dopamine concentrations but has no serotonergic effects [16], and does not increase cortisol levels [11]. Thus, both MDMA and MPH share sympathomimetic and psychostimulant effects, yet with distinct hormone profiles.

Here we used drug-induced stress to test its effects on the human immune system. Specifically, we studied stress hormone-mediated recruitment of NK cell subsets in the context of a randomized, double-blind, placebo-controlled clinical trial of subjects receiving MDMA and/or MPH.

## Materials and Methods

### Clinical study

Healthy subjects (n = 12, mean age 24.9 years, 8 female, 4 male) from a pharmacological study (NCT01465685) on the effects of single-dose MDMA (125 mg), MPH (60 mg) or a combination of the two drugs were tested for the effects of the drugs on the immune system. Characteristics of the study and the participants have previously been published [12]. All subjects provided written informed consent. The study was approved by the ethics committee of the canton of Basel, Switzerland (EKBB 228/11) and conducted in accordance with the Declaration of Helsinki. All subjects received each drug condition once in a double-blind, placebo-controlled, crossover design with four experimental test sessions (placebo–placebo, MPH–placebo, placebo–MDMA, and MPH–MDMA). Drugs were taken orally. Washout periods between two conditions were at least 10 days. All blood draws were performed via an indwelling intravenous catheter placed in an antecubital vein and the baseline blood draw was at 8 am for all study subjects. Peripheral blood mononuclear cells (PBMC) were isolated using Lymphoprep^TM^ (Axis-Shield, Oslo, Norway) density centrifugation and cryopreserved until use, in order to be able to measure all study timepoints (i.e. conditions) for each subject on the same day. The cardiovascular stress response (systolic blood pressure and heart rate) was repeatedly recorded and plasma levels of norepinephrine, epinephrine, dopamine and cortisol were determined before and 2h after drug administration [12].

### Flow cytometry based immunophenotyping

PBMC were immunophenotyped using the following antibodies: CD3-PerCP (UCHT1), CD4-APC (OKT4), CD18-PE (TS1/18), CD19-PE (HIB19), NKG2D-APC (1D11), CX3CR1-FITC (2A9-1, all Biolegend), CD3-Pacific Blue (UCHT1), CD8-FITC (HIT8a), CD16-PE-Cy5 (3G8), CD56-PE, -Alexa Fluor 647 or -PE-Cy7 (B159), CD69-FITC (FN50) (all BD Biosciences), CD62L-FITC (LT-TD180) (ImmunoTools), GCR-Alexa Fluor 488 (D8H2) (Cell Signaling Technology) and ADRB2 pure (S364, RayBiotech). The antibody against ADRB2 was coupled to R-phycoerythrin (RPE) in house using the ABSelect BSA Removal Kit to clean and concentrate the pure ADRB2 antibody followed by fluorochrome coupling with the Lightning-Link® R-Phycoerythrin Conjugation Kit according to manufacturer’s instructions (Innova Biosciences).

All stainings were performed as surface-stainings for 30 min. at 4°C, with exception of the intracellular GCR staining that was performed in whole blood according to a standard ICS protocol (Cell Signaling Technology). All sample acquisitions were performed on an Accuri^TM^ C6 (immune cell subsets and NK function) or a LSRFortessa^TM^ (NK receptor phenotyping) flow cytometer (BD Biosciences).

### NK cell *in vitro* stimulation assays

NK cell function was assessed by IFNγ production and degranulation (CD107a) as an indirect marker of cytotoxicity [17]. NK cells were stimulated with K562 cells by co-culture of 10^6^ PBMC at a 10:1 effector-to-target ratio as previously described [18]. Following 30 min. of pre-incubation, cells were incubated during 4 hours in the presence of Brefeldin A (Biolegend) at 0.5 μg/mL, Monensin (Golgi-Stop^TM^, BD Biosciences) at 0.3 μg/mL and anti-CD107a-APC (H4A3) (BD Bioscience). Cells were then surface-stained using anti-CD3 PerCP and anti-CD56 PE followed by intracellular staining for IFNγ using Cytofix/Cytoperm^TM^ (BD Biosciences) and anti-IFNγ-FITC (4S.B3) (Biolegend). For experiments testing the impact of stress hormones on NKG2D expression *in vitro*, cells were incubated for 3 h in serum-free RPMI 1640 (Life Technologies) in the presence of cortisol (Sigma) at 280 ng/mL, epinephrine (Sintetica) at 50 pg/mL, or a combination of both and NKG2D mean fluorescence intensity (MFI) was assessed.

### Hormone receptor quantification on NK cell subsets using real-time RT PCR

The β2-adrenergic receptor (ADRB2) and the glucocorticoid receptor (GCR) expression on NK cell subsets were quantified by real-time PCR. CD56^bright^ and CD56^dim^ NK cells were sorted on a FACSAria^TM^ III (BD Bioscience) and RNA was extracted using the QIAamp® RNA Blood Mini Kit (Qiagen). Genomic DNA was removed and first-strand cDNA was generated by means of GoScript^TM^ Reverse Transcription System (Promega). RT-PCR was performed with GoTaq® qPCR Master Mix in duplicates in a 7500 Fast Real-Time PCR System (Applied Biosystems) using the following primers (Microsynth, Switzerland): ADRB2: 5’-ACAGGGGAGCAGAGTGGATA-3’ and 3’- ACAGTACCTTGATGGCCCAC-5’ and GCR: 5’-TGGGGACTCTGAACTTCCCTG-3’ and 3’- CTGTTGTTGCTGTTGAGGAGC-5’ (Complete primer list available in **S1 Protocol**). The amplification process consisted of polymerase activation at 95°C for 10 min., 45 cycles with 15 s of denaturation at 95°C and 1 min. of annealing and elongation at 60°C and a final elongation step for 1 min. at 60°C. For all samples signals were detected between cycles 24 and 35. Two reference genes (GADPH and PKG1) were used for relative quantification. A more detailed description of the qPCR protocol is available in **S1 Protocol**.

### Data analysis and statistics

All flow cytometry data was analyzed blinded for the drug condition using FlowJo Software (Version X 10.0.7). Gating strategies for the analyses are shown in the supporting information (**S2 Fig** and **S3 Fig**). Statistical analyses and data visualization was performed using Prism software (Prism 6.0, Graphpad). Group comparisons were performed using t-tests or Kruskal Wallis tests followed by Dunn’s comparisons. Correlation analyses were performed using Spearman Rank tests.

## Results and Discussion

### Drug-induced cardiovascular stress responses associate with an increased proportion of peripheral Natural killer cells

**Fig 1:**
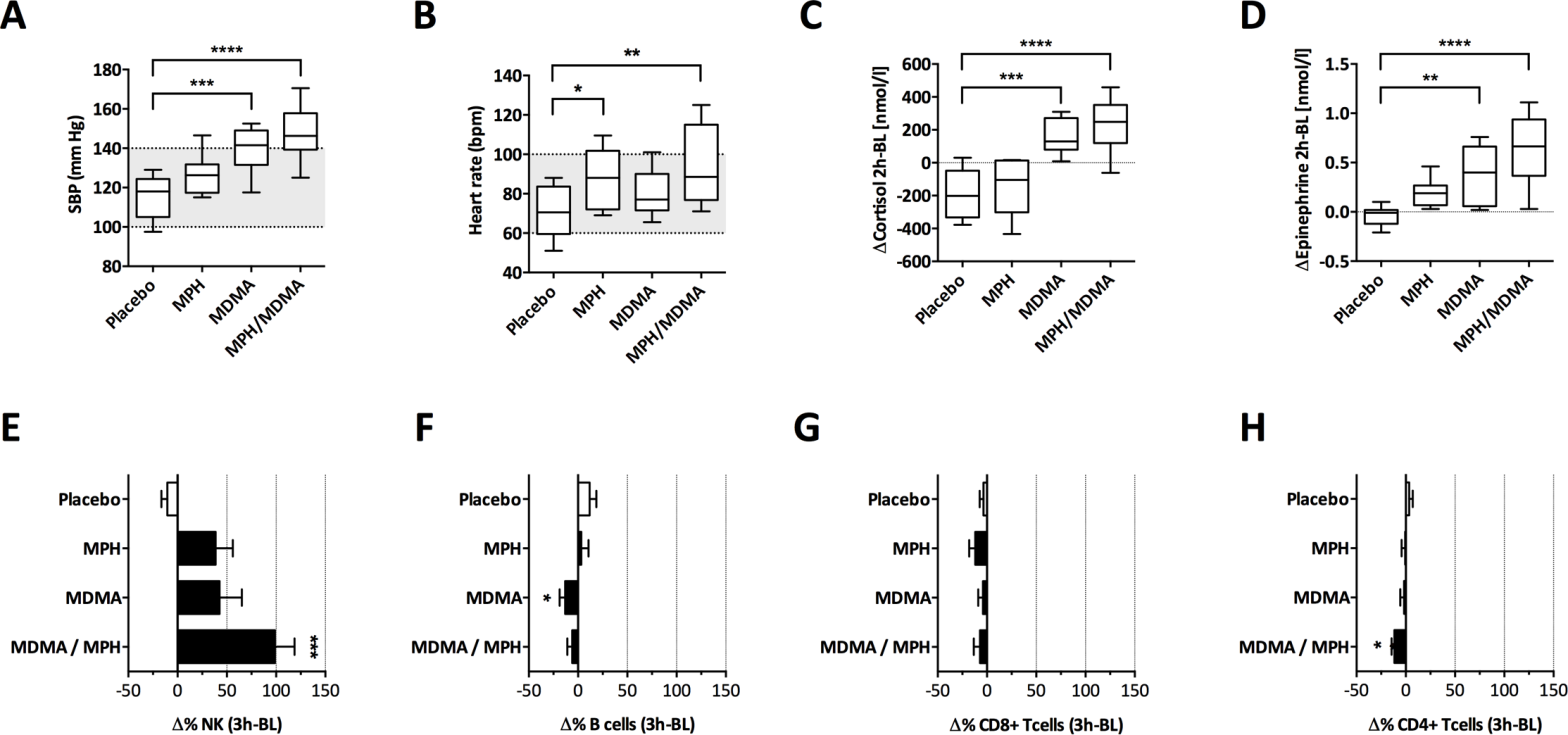
Drug-induced stress induces a pronounced cardiovascular responses and NK cell recruitment. (A) Systolic blood pressure (SBP) in mmHg and (B) heart rate (HR) in beats per minute (bpm) are shown three hours (i.e. one hour after the stress hormone peak) post-adminstration of the study drug (n = 12). The grey shaded area indicates the normal values. Serum cortisol (C) and epinephrine (D) levels were measured before and two hours post drug administration and the difference from baseline was calculated. Box plots indicate median and IQR, whiskers indicate range. (E-H) Lymphocyte subset distribution 3 hours after drug administration was examined (n = 12). Percent change from baseline level to three hours post administration (Δ%) was calculated for each lymphocyte subset. Mean Δ% and S.E.M is shown for the four main lymphocyte subsets: (E) NK cells (CD3^-^ CD56^+^), (F) B cells (CD3^-^ CD19^+^), (G) CD8 cytotoxic T cells (CD3^+^ CD8^+^) and (H) CD4 T helper cells (CD3^+^ CD4^+^). Kruskal-Wallis test with Dunn’s correction to test for multiple comparisons was used (*p<0.05, **p<0.01, ***p<0.001, ****p<0.0001).

The cardiovascular response and stress hormone levels in the peripheral blood can indicate stress. MDMA, MPH, and the combination of the two drugs induced an increase in systolic blood pressure (SBP) and heart rate peaking three hours after drug administration (**Fig 1A and 1B**). Details to the cardiovascular response in the study subjects have been reported previously [12]. The MDMA/MPH combination induced the strongest increase in SBP, and MPH alone induced a SBP increase of lower magnitude than the conditions that included MDMA (**Fig 1A**). Contrarily, the heart rate was highest in treatment conditions including MPH (**Fig 1B**). Since these differences might be linked to different stress hormone profiles induced by the different treatments, levels of cortisol and catecholamines were analyzed and compared with levels measured in placebo treated study subjects. Hormone levels two hours after drug administration (i.e. at their peak) were compared to the baseline blood draws, which were all performed at 8 am. All active drug conditions resulted in a comparable, albeit non-significant increase of norepinephrine at 2 hours (*data not shown*). The most striking difference between the drug conditions was an increase in cortisol concentration upon exposure to MDMA (p < 0.001) or MDMA/MPH (p < 0.0001) compared to placebo or MPH alone (**Fig 1C**). Cortisol levels fell in the placebo and MPH condition, consistent with known circadian changes in cortisol, given the timing of the blood draws, all starting at 8 am [19]. The increase in epinephrine concentrations was higher in individuals exposed to MDMA/MPH (p < 0.0001) than in those exposed to MDMA alone (p < 0.01) and lowest in participants after MPH intake alone, suggesting an additive effect of MDMA and MPH on the epinephrine stress response (**Fig 1D**). The cardiovascular stress response (i.e. HR increase and SBP increase) correlated with both, the epinephrine (HR: p = 0.0035, r = 0.42; SBP: p = 0.0001, r = 0.53), and cortisol increase (HR: p = 0.0001, r = 0.53; SBP p<0.0001, r = 0.71). Together, the findings suggest that the study conditions resulted in distinct cardiovascular and endocrine stress response profiles.

Acute stress has been linked to a recruitment of NK cells to the peripheral blood in various models [6]. To test whether the same applies to pharmacological stress induced by MDMA, MPH or their combination, we monitored the change in lymphocyte subset-distribution at baseline and three hours after drug administration. This time-point was chosen based on the stress hormone level peak that occurs around 2–3 hours after drug-intake [11]. We then calculated the percentage change in T cells (CD4+ and CD8+), B cells and NK cells compared to the baseline distribution (gating strategy is depicted in **S2 Fig**). The most striking observation was a dramatic increase of NK cells within the lymphocyte compartment that was most pronounced upon exposure to combination treatment of MDMA and MPH (p < 0.001 **Fig 1E**). The other immune cell subsets were variably decreased (**Fig 1F–1H**). Pacifici *et al*. also reported a comparable and rapid increase of total NK cells in MDMA treated subjects, which was however not further investigated [14, 20–22]. To enable an immediate response to any threat it is plausible that early stress-induced adaptations act through cell redistribution, and preferentially impact innate immunity that can unfold much more rapidly [6, 23].

Because this rapidly occurring redistribution of lymphocyte subsets suggested stress hormone-mediated effects, we next correlated stress hormone levels (2 hours post administration) with the individual NK cell increase, irrespective of the treatment. NK cell increase correlated with the change in epinephrine levels (p = 0.0049, r = 0.44; **S1A Fig**), but not with the change of cortisol or norepinephrine (**S1B and S1C Fig**). Dopamine was only very weakly (median 0.03 nmol/L, IQR -0.03–0.09) and inconstantly increased following any drug administration and the change in dopamine levels showed no correlation with the change in NK cell frequencies (data not shown). Similarly, we found no correlation between drug induced neuropsychological effects -namely changes in activity or emotional excitability- and NK cell redistribution. Taken together, this hinted at a more pronounced role of epinephrine in NK cell redistribution.

### Stress enriches for circulating cytotoxic NK cells with unaltered effector functions and activation state

**Fig 2:**
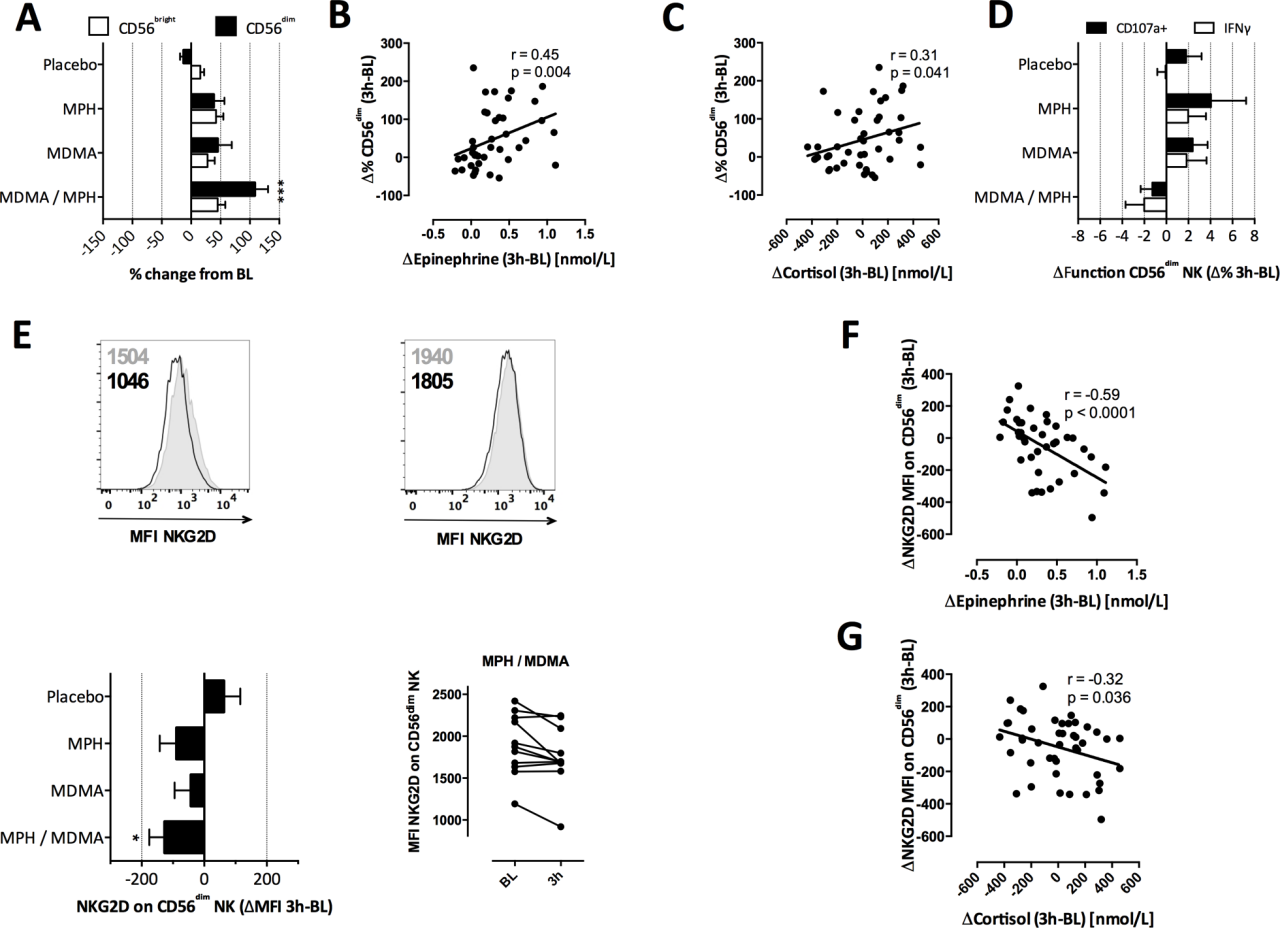
Unaltered NK function but reduced NKG2D expression upon drug-induced stress. (A) Percent change in the two NK cell subsets was calculated and expressed as mean + S.E.M. (B,C) The percent change of CD3^-^ CD56^dim^ NK cells was correlated to the change in serum epinephrine (B) and cortisol (C) levels 2 hours p.a. in all drug categories together (n = 44). (D) NK function against K562 target cells was assessed. Changes in IFNγ^+^ or CD107a^+^ (degranulation) CD56^dim^ NK cells are shown for each drug category as absolute change in percentage (n = 11, mean + S.E.M). (E) Example histograms of NKG2D MFI before (grey) and after (black) MPH / MDMA treatment are shown for a donor with a strong (left) and weak (right) response (upper panels). The numbers indicate the MFI. Absolute change in the MFI of NKG2D expression (n = 12, mean + S.E.M) is displayed for each drug condition (lower left panel). NKG2D MFI at baseline and 3 hours p.a. is shown separately for all subjects for the condition with a significant difference to placebo (MPH / MDMA) (lower right panel). The absolute change in MFI of NKG2D was correlated to serum epinephrine (F) and cortisol (G) levels irrespective of drug treatment (n = 44). Spearman correlation and nonlinear fit were applied for correlation analyses and Kruskal-Wallis test was applied for all group comparisons. *P < 0.05, **P < 0.01, ***P < 0.001.

Given their strong relative increase upon drug-induced stress, we performed a more detailed analysis of the specific NK cell subsets and their functionality. Although both cytotoxic (CD56^dim^) and immunomodulatory (CD56^bright^) NK cell subsets increased, the most pronounced effect was detected for CD56^dim^ NK cells upon the combined drug stimulation (**Fig 2A**). This indicated that CD56^dim^ NK cells were recruited preferentially upon stress. The stronger correlation between the change in CD56^dim^ NK cells and the alteration in epinephrine levels (p = 0.004, r = 0.45, **Fig 2B**), as compared to the correlation with change in cortisol levels (**Fig 2C,** p = 0.041, r = 0.31), further supported this notion. In contrast, we did not detect any correlation of CD56^bright^ NK cells and hormone levels (*data not shown*). Interestingly, the shift in the NK cell subsets did not translate into enhanced NK effector functions against target cells (**Fig 2D**). Previous studies variably associated acute stress with enhanced [6], or decreased NK cell activity [24, 25], which could relate to different assay conditions (e.g. not including human plasma in the culture media, cryopreservation [26]), some of which were, however, inevitable in order to test all samples from a donor simultaneously with the same target cell conditions. Reduced function has moreover been attributed to glucocorticoid-induced epigenetic changes, which we might have missed by testing NK function within three hours post-stressor [27].

Drug-induced stress consistently associated with a moderate, but significant lower per-cell expression (i.e. mean fluorescence intensity; MFI) of NKG2D, an NK cell activating receptor recognizing stress-induced proteins (**Fig 2E**). This decrease was limited to CD56^dim^ NK cells, and NKG2D on bulk T cells was slightly increased (*data not shown*). NKG2D expression on CD56^dim^ NK cells was inversely correlated with the change in epinephrine levels (**Fig 2F**, p < 0.0001, r = -0.59), compared to an again more moderate effect of cortisol (**Fig 2G**, p = 0.036, r = -0.32). This NKG2D^low^ phenotype might reflect a safety measure preventing an overshooting NK stimulation that would result in excessive immunopathology [28–31]. Due to sample availability, we could however not address this hypothesis experimentally. Treatment of NK cells with synthetic epinephrine and cortisol *in vitro* dampened NK function, as expected from the literature [32]. Yet, this NK cell inhibition was independent of NKG2D expression, as NKG2D was only reduced inconstantly (i.e. six of eleven healthy donors) and showed no correlation with NK function. Moreover, a reduction in NK function was also observed against 721.221 target cells not expressing NKG2D ligands (data not shown). Together, this indicates that the NKG2D^low^ phenotype results from mobilization of these NK cells rather than from a receptor down-modulation by direct hormone effects.

### Inherent expression differences of key surface receptors may explain preferential recruitment of CD56^dim^ NK cells

**Fig 3:**
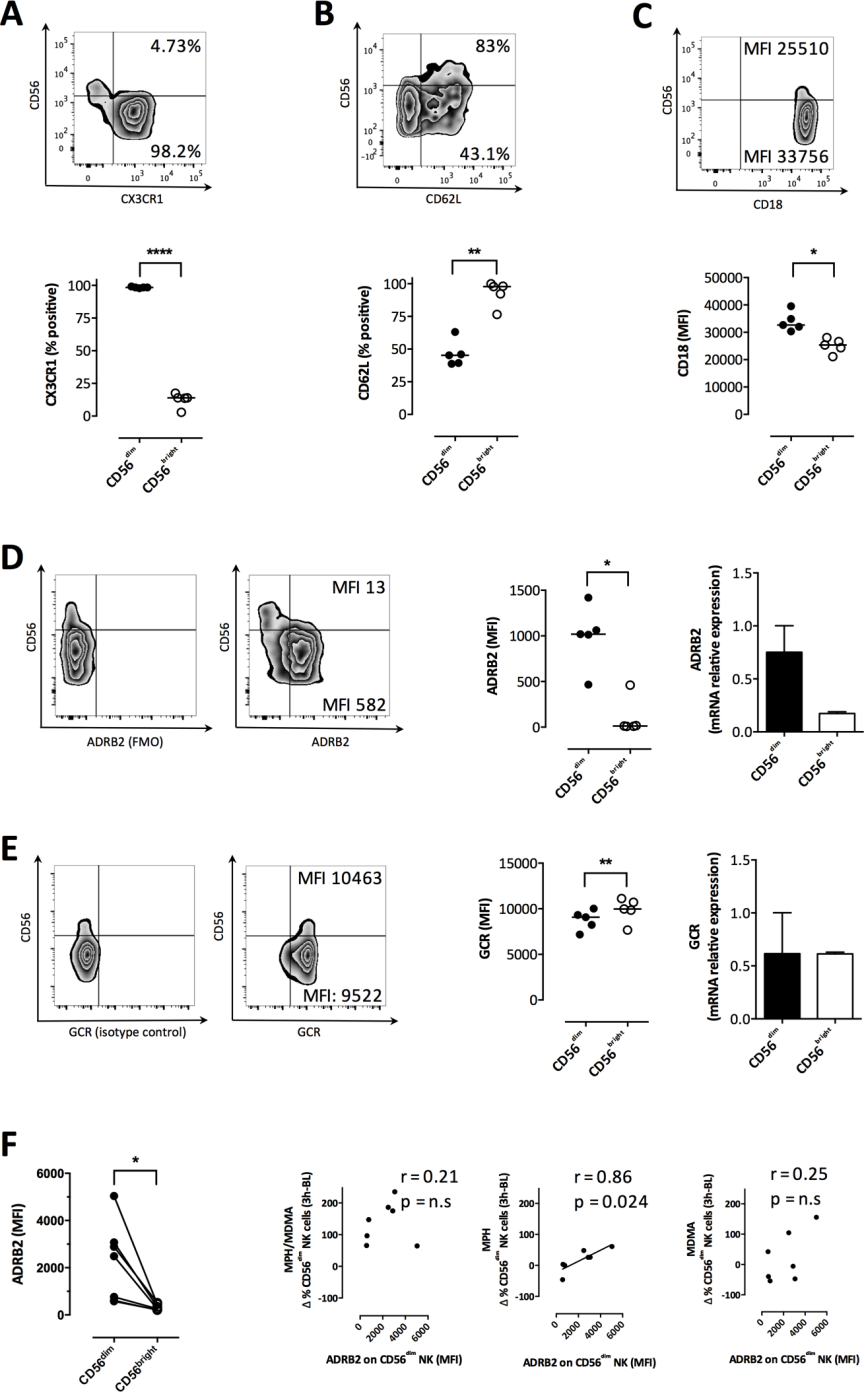
Preferential recruitment of CD56^dim^ NK cells depends on distinct hormone and migration receptor expression profiles. Expression of three receptors important in cell adhesion and migration were assessed by flow cytometry. (A-C) Example stainings gated on CD3^-^ CD56^+^ lymphocytes are shown for CX3CR1 (A), CD62L (B) and CD18 (C). The frequency (A, B) or MFI (C) of receptor expression on CD56^bright^ and CD56^dim^ is indicated (upper panels). Data on freshly derived PBMCs from five healthy, unstressed donors are summarized (A-C lower panels). (D, E) Expression of the epinephrine receptor (ADRB2) (D) and the glucocorticoid receptor (GCR) (E) were assessed on CD3^-^ CD56^+^ lymphocytes by flow cytometry. Isotype control stainings for ADRB2 and GCR are shown, followed by an example staining (left panels). The MFI of the two subsets is indicated for CD56^bright^ (upper right corner) and CD56^dim^ NK cell subsets (lower right corner). Flow cytometric data of the aforementioned five donors is summarized for CD56^bright^ and CD56^dim^ NK cells (middle panels). Differential ADRB2 and GCR expression on the two NK cell subsets were also confirmed on mRNA level by measuring relative hormone receptor expression on sorted CD56^dim^ (n = 3) and CD56^bright^ NK cells (n = 2) by qPCR (right panels). (F) Differential expression of ADRB2 on CD56^bright^ and CD56^dim^ NK cells was confirmed in study participants 1 hour after placebo treatment (left panel, n = 7). ADRB2 expression was correlated to the increase in NK cell percentage upon drug administration. Paired t tests were applied for all group comparisons and Spearman correlation and nonlinear fit for correlation analyses. *P < 0.05, **P < 0.01, ****P < 0.0001.

Our immunophenotypic data suggested that drug-induced stress associated with the preferential recruitment of cytotoxic NK cells without substantially affecting their function. Thus, we next aimed at defining molecular differences between CD56^dim^ and CD56^bright^ NK cell subsets that may explain the preferential recruitment of the CD56^dim^ subset. First, we assessed the expression-profile of receptors previously linked to epinephrine-mediated lymphocyte redistribution, namely high expression of the fractalkine receptor CX3CR1 and low expression of CD62L [33], in five healthy untreated individuals. The flow cytometry gating strategies are shown in the supporting information (**S3 Fig**). Indeed, we found that the CD56^dim^ subset expressed very high levels of CX3CR1 (**Fig 3A**), but low levels of CD62L (**Fig 3B**). This expression profile was inverse in the CD56^bright^ subset (CX3CR1, p < 0.0001; CD62L, p = 0.001). In contrast, CD18, an integrin known to bind to ICAMs and thereby enhancing NK cell migration [34] was slightly more expressed on the CD56^dim^ NK subset (p = 0.008, **Fig 3C**). Next, we assessed mRNA and protein levels of the β2-adrenergic receptor (ADRB2) and glucocorticoid receptor (GCR), i.e. the receptor for epinephrine and cortisol respectively, in the two NK cell subsets. Both qPCR- and flow cytometry-based quantification indicated higher expression of the ADRB2 among CD56^dim^, compared to the CD56^bright^, NK cells (p = 0.019 for MFI, **Fig 3D**). In contrast, the GCR was lower expressed in CD56^dim^ NK cells (p = 0.005 for MFI, **Fig 3E**). Taken together, and in line with the correlation analyses of the *in vivo* data, this was suggestive that the CD56^dim^ subset was able to respond more vigorously to epinephrine.

Interestingly, we observed that ADRB2 abundance varied considerably between individuals (**Fig 3D**). Therefore, we next tested whether the individual ADRB2 receptor expression on NK cells might have determined the increase of NK cells *in vivo* in the clinical study. We first confirmed the inter-individual and NK subset-specific variability of ARDB2 expression in seven subjects included in the clinical stress study (**Fig 3F**). Importantly, the individual ADRB2 expression was stable over time, as the same donors showed comparable levels on separate study days (*data not shown*). Taking advantage of the distinct hormone changes induced by the different drug conditions (**Fig 1C and 1D**), epinephrine effects were analyzed by correlating the change in NK cells and altered ADRB2 expression in the presence (MDMA or MDMA/MPH group) or absence (MPH) of a concomitant cortisol increase. We found a very strong correlation between an increase of NK cells and ADRB2 expression in the MPH group (p = 0.02, r = 0.86), i.e. in absence of a cortisol response. Similar trends, albeit much weaker, were seen in the other groups (**Fig 3F**), suggesting that cortisol might contribute to NK cell distribution in these conditions.

Together, our findings pointed at an important role of epinephrine in the early changes in the NK cell compartment. Interestingly, in the absence of a cortisol response and at low epinephrine levels (i.e. the MPH treatment condition), NK cell redistribution correlated almost perfectly with the expression level of the beta-adrenergic receptor. This is in line with a model of selective catecholamine-dependent regulation of NK cell adhesion to the endothelia [24], as supported by studies using beta-adrenergic blockade suggesting catecholamines as master regulators of early stress effects on the immune system [35].

## Conclusion

Stress was observed to induce an enrichment of CD56^dim^ NK cells in the peripheral blood, which associated with a strong epinephrine response. These NK cells expressed a receptor profile that would enable them to rapidly re-distribute to a site of injury or infection, where this cytotoxic subset could contribute to pathogen defense and wound healing. Differences in hormone receptor expression might explain differences in the magnitude of individual stress responses. The insights gained from this controlled clinical trial setting, using standardized stressors that consistently induced distinct stress hormone profiles, support that, in humans, NK cells can be recruited through epinephrine dependent mechanisms, which may be beneficial e.g. in leukemia or cancer therapy [36]. Our study thus adds a rationale to interventions that increase endogenous epinephrine levels (i.e. physical activity, MDMA etc…) currently explored e.g. as adjunctive cancer treatments.

## Supporting Information

S1 Fig Correlation of NK cell increase with serum hormone levels. Absolute change in NK percentage 3 hours after drug administration was correlated to change in concentrations of epinephrine (A), cortisol (B) and norepinephrine (C) 2 hours post treatment. Spearman correlation and nonlinear fit were applied.
(TIFF)

S2 Fig Gating strategy to define lymphocyte and NK cell subsets and to assess NKG2D expression and IFNγ and CD107a production in NK cell subsets. The top row shows the gating in order to distinguish B cells, CD4+ and CD8+ T cells. A second panel was used to detect NK cells, further subdividing them into CD56 dim and CD56 bright NK cells and analyzing IFNγ and CD107a production (**2**, middle row, data is shown for CD56 dim). A third panel was designed to detect NKG2D expression (**3**, CD314, bottom middle) and a last one to stain for the glucocorticoid receptor (**4**, bottom right) on NK subsets. In grey, full stainings are shown whereas in white, an FMO staining is depicted for NKG2D and an isotype control for GCR. All data was acquired on an Accuri C6 (Becton Dickinson).
(TIFF)

S3 Fig Gating strategy for the analysis of adhesion and hormone receptor expression on NK cell subsets. NK cells were defined by CD16 and CD56 expression on single, live CD3- cells (upper row). The first panel stained for ADRB2 and CD62L on NK cell subsets whereas the second panel stained for CX3CR1 and CD18. Example stainings on CD56dim NK cells are shown in dark grey while isotype control (ADRB2) or fluorescence minus one controls (FMO, for CD62L, CX3CR1 and CD18) are shown in light grey. All data was acquired on an LSRFortessa flow cytometer (Becton Dickinson).
(TIFF)

S1 Protocol Detailed RT-PCR protocol for the hormone receptor analysis incl. a complete list of the primers used. (DOCX)
