# Supplementary figures and images for: Stress-Induced In Vivo Recruitment of Human Cytotoxic Natural Killer Cells Favors Subsets with Distinct Receptor Profiles and Associates with Increased Epinephrine Levels
Source: PLoS One. 2015 Dec 23;10(12):e0145635. doi: 10.1371/journal.pone.0145635 (PMC4689586; doi:10.1371/journal.pone.0145635)

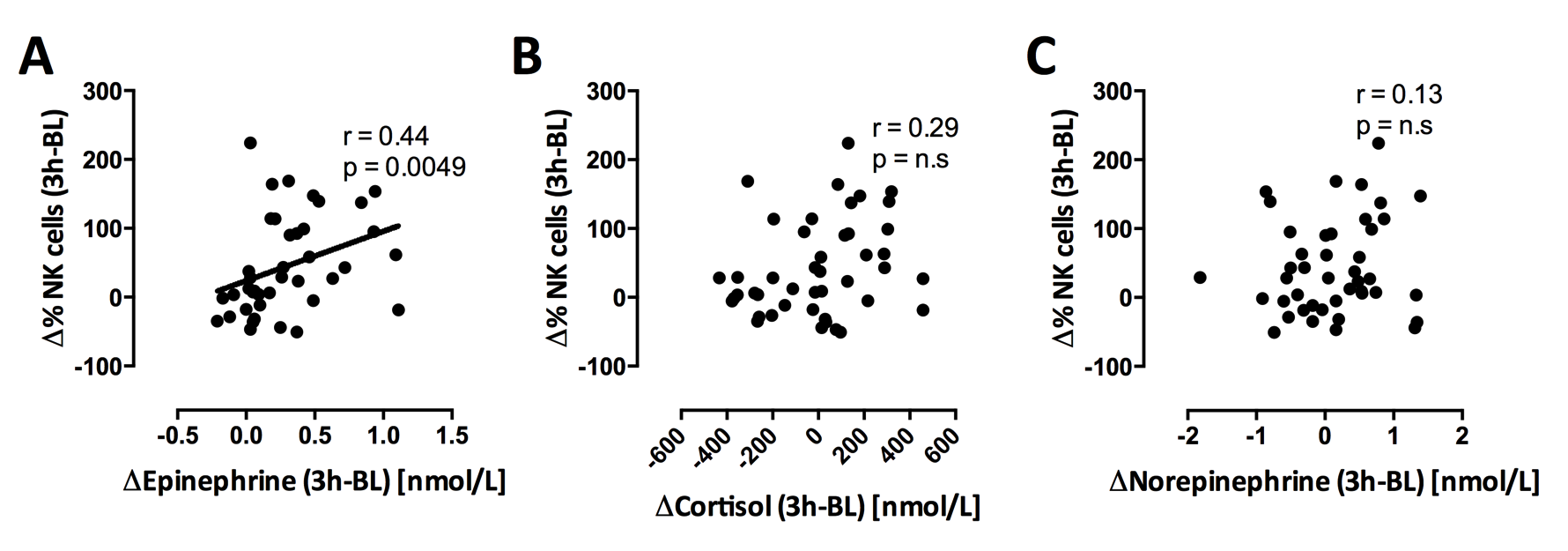

Supplement: S1 Fig — Absolute change in NK percentage 3 hours after drug administration was correlated to change in concentrations of epinephrine (A), cortisol (B) and norepinephrine (C) 2 hours post treatment. Spearman correlation and nonlinear fit were applied. (TIFF) [file pone.0145635.s001.tiff]

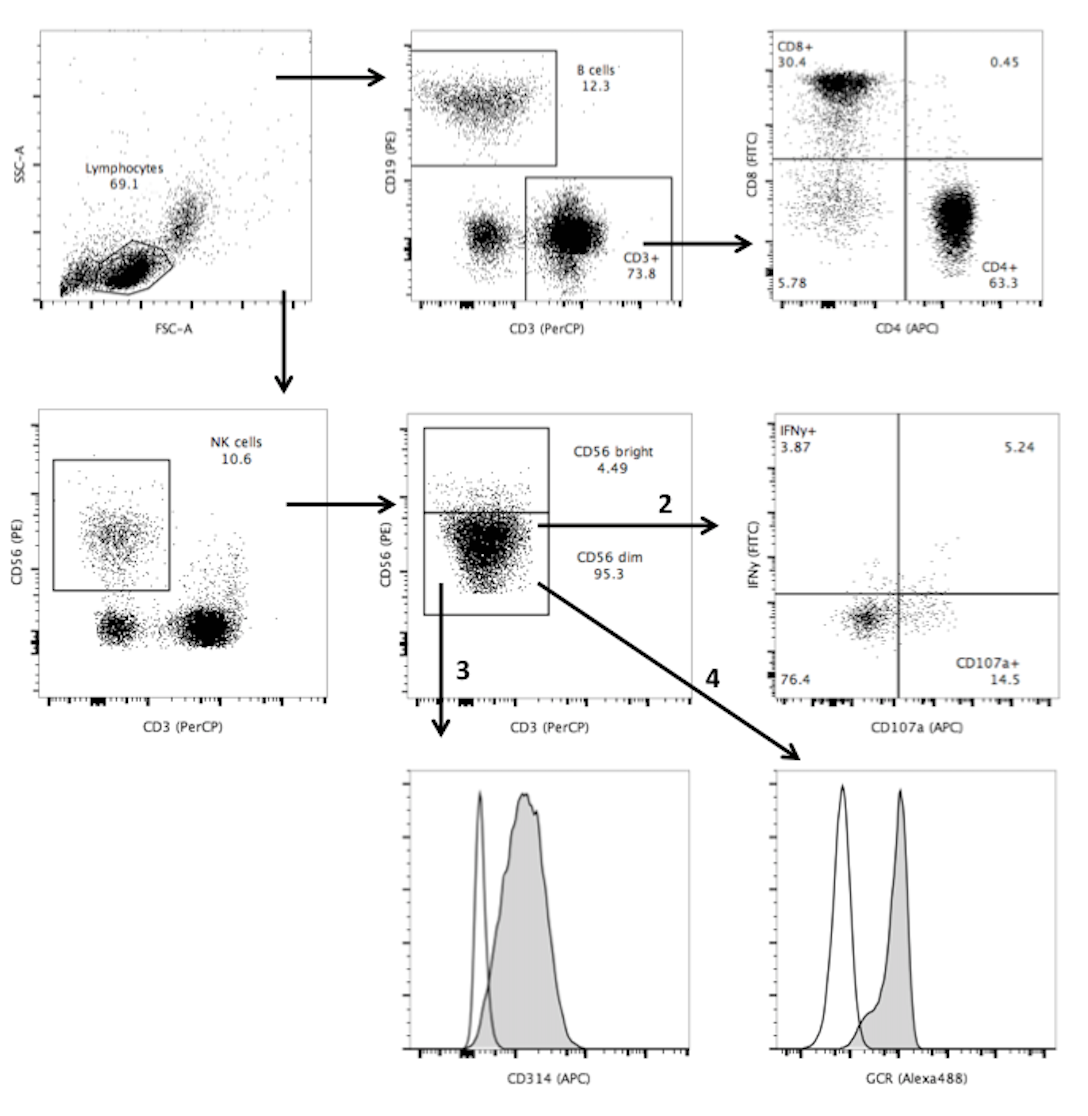

Supplement: S2 Fig — The top row shows the gating in order to distinguish B cells, CD4+ and CD8+ T cells. A second panel was used to detect NK cells, further subdividing them into CD56 dim and CD56 bright NK cells and analyzing IFNγ and CD107a production (2, middle row, data is shown for CD56 dim). A third panel was designed to detect NKG2D expression (3, CD314, bottom middle) and a last one to stain for the glucocorticoid receptor (4, bottom right) on NK subsets. In grey, full stainings are shown whereas in white, an FMO staining is depicted for NKG2D and an isotype control for GCR. All data was acquired on an Accuri C6 (Becton Dickinson). (TIFF) [file pone.0145635.s002.tiff]

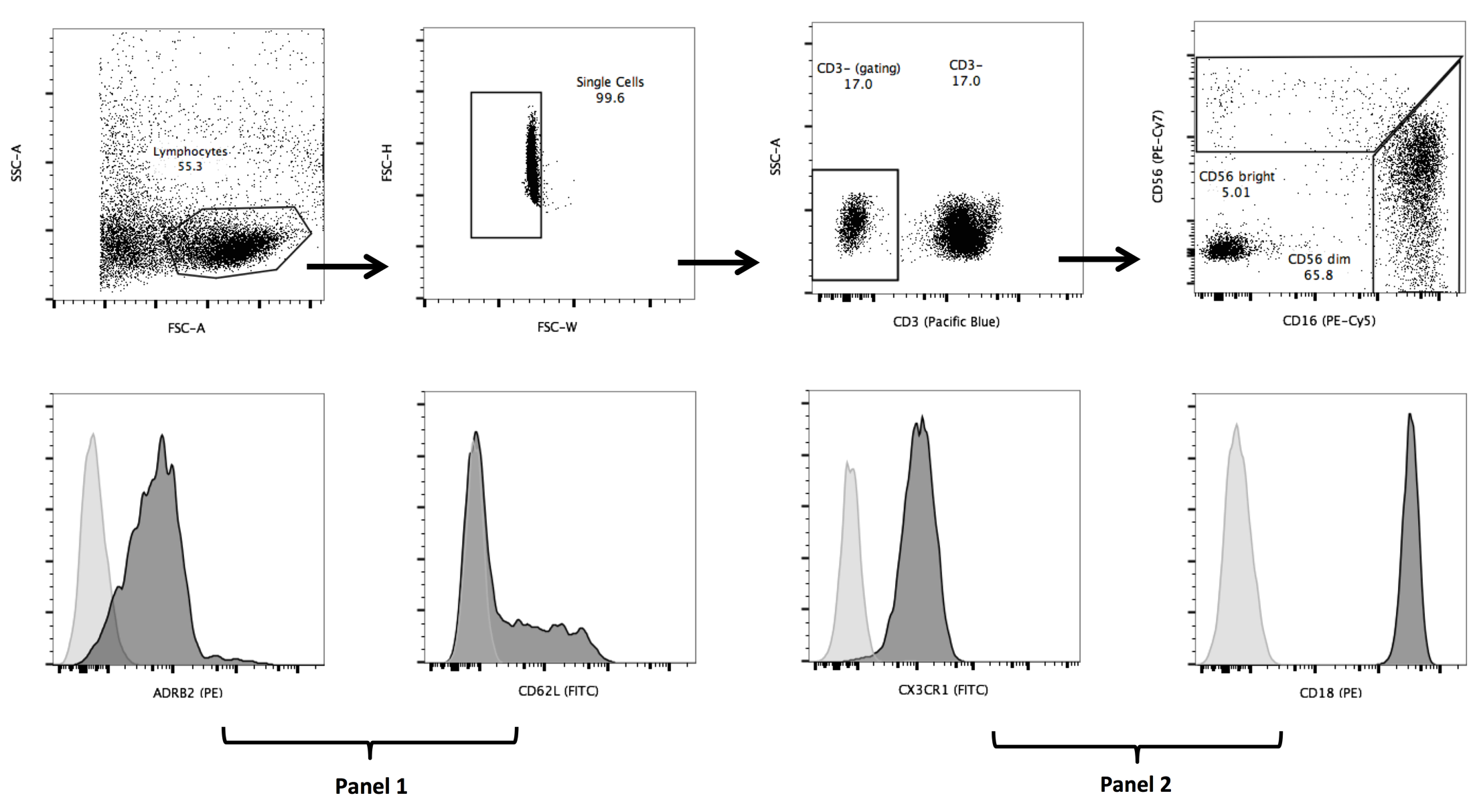

Supplement: S3 Fig — NK cells were defined by CD16 and CD56 expression on single, live CD3- cells (upper row). The first panel stained for ADRB2 and CD62L on NK cell subsets whereas the second panel stained for CX3CR1 and CD18. Example stainings on CD56dim NK cells are shown in dark grey while isotype control (ADRB2) or fluorescence minus one controls (FMO, for CD62L, CX3CR1 and CD18) are shown in light grey. All data was acquired on an LSRFortessa flow cytometer (Becton Dickinson). (TIFF) [file pone.0145635.s003.tiff]
