# Supplementary material for: Stress-Induced In Vivo Recruitment of Human Cytotoxic Natural Killer Cells Favors Subsets with Distinct Receptor Profiles and Associates with Increased Epinephrine Levels
Source: PLoS One. 2015 Dec 23;10(12):e0145635. doi: 10.1371/journal.pone.0145635 (PMC4689586; doi:10.1371/journal.pone.0145635)
Supplement: S1 Protocol — (DOCX) [file pone.0145635.s004.docx]

**SUPPLEMENTARY METHODS**

**Primers for real-time RT PCR**

All primers were designed with Primer3 software in a way to create amplicons spanning exon-exon junctions wherever possible (except for ADRB2 and ACTB). We also aimed for maximal isoform coverage with help of the Ensembl database. Primers were ordered at Microsynth Switzerland.

| **Symbol** | **Forward primer** | **Reverse Primer** | **Product length** |
| --- | --- | --- | --- |
| ADRB2 | ACAGGGGAGCAGAGTGGATA | ACAGTACCTTGATGGCCCAC | 104 bp |
| GCR | TGGGGACTCTGAACTTCCCTG | CTGTTGTTGCTGTTGAGGAGC | 111 bp |
| ACTB | CGAGCACAGAGCCTCGCCTT | CATCATCCATGGTGAGCTGGCG | 70 bp |
| GADPH | TCTTCTTTTGCGTCGCCAGCC | CCCAATACGACCAAATCCGTTGA | 87 bp |
| PKG1 | GTTGACCGAATCACCGACCT | GTCGACTCTCATAACGACCCG | 115 bp |
| HPRT | ATGGACAGGACTGAACGTCT | TCCAGCAGGTCAGCAAAGAA | 113 bp |

**Hormone receptor expression using real-time RT PCR**

NK cells were enriched from PBMC using the magnetic beads based NK enrichment kit (Miltenyi Biotec) according to the manufacturer’s instructions. CD56^bright^ and CD56^dim^ NK cells were then sorted on a FACSAria III (BD Bioscience) and RNA was extracted using the QIAamp RNA Blood Mini Kit (Qiagen). Up to 1 μg of extracted RNA was treated with 1 unit of DNAse I (Promega) to remove genomic DNA. Immediately afterwards, first-strand cDNA was generated by means of GoScript^TM^ Reverse Transcription System according to manufacturer’s protocol (Promega) using random hexamer primers and a MgCl_2_ concentration of 3 mM. RT-PCR reactions (20 μl) contained variable amounts of template cDNA (5 μl), 2x GoTaq Probe qPCR Master Mix (10 μl), nuclease-free water (3 μl) and 500 nM forward and reverse primers each (2 μl). Reactions were run in duplicates in a 7500 Fast Real-Time PCR System (Applied Biosystems). The amplification process consisted of polymerase activation at 95°C for 10 minutes, 45 cycles with 15 s of denaturation at 95°C and 1 minute of annealing and elongation at 60°C and a final elongation step for 1 minute at 60°C. Specificity of all primer pairs was confirmed with melt curves, efficiencies (E) were calculated with a standard curve of four serial dilution points according to the following formula:

$$E= {10}^{(-\frac{1}{slope})}$$

For normalization, suitable reference genes had to be found. This was done by application of the GeNorm algorithm [40] for assessing the most stable expression in four potential reference genes in five samples. The two best reference genes (GADPH and PKG1) were used for relative quantification by application of the following formula:

$$NRQ= \frac{E^{\Delta Cq, goi, goi}}{\sqrt{E^{\Delta Cq,GADPH,GADPH}*E^{\Delta Cq, PKG1,PKG1}}}$$

where NRQ is normalized relative quantity, Cq is cycle of quantification and goi is gene of interest.
